# Epidemiology and Healthcare Utilization in Pediatric Multiple Sclerosis and Neuromyelitis Optica: A Nationwide Population-Based Study in South Korea (2016–2020)

**DOI:** 10.3390/children11050553

**Published:** 2024-05-05

**Authors:** Hyewon Woo, Junho Hwang, Sun Ah Choi, Soo Ahn Chae

**Affiliations:** 1 Department of Pediatrics, Chungbuk National University Hospital, Cheongju 28644, Republic of Korea; c303014@cbnuh.or.kr; 2 Department of pediatrics, Chung-Ang University Hospital, Chung-Ang University College of Medicine, Seoul 06973, Republic of Korea; 789654jun0@naver.com (J.H.); kidbrain@cau.ac.kr (S.A.C.); 3 Department of pediatrics, Ewha Womans University Mokdong Hospital, Ewha Womans University College of Medicine, Seoul 07985, Republic of Korea

**Keywords:** multiple sclerosis, neuromyelitis optica, acute demyelinating syndrome, pediatrics, epidemiology, health services research

## Abstract

Pediatric multiple sclerosis (MS) and neuromyelitis optica (NMO) are rare acquired demyelinating syndrome with limited epidemiological data available, particularly in non-Western setting. This study aimed to demonstrate the epidemiology of pediatric MS and NMO in South Korea and to analyze of healthcare utilization and economic burden associated with these conditions. Using a nationwide population-based database from the Korean Health Insurance Review and Assessment Service database, we identified pediatric cases (age < 20 years) of MS and NMO from 2016 to 2020. We analyzed incidence, prevalence, healthcare utilization and medical costs. The study found low age-standardized incidence and prevalence rates for pediatric MS and NMO in South Korea. There was a marked disparity in healthcare utilization between urban and rural areas. Most healthcare interactions occurred in tertiary hospitals in urban settings, particularly in Seoul. The study also highlighted the substantial economic burden associated with the management of rare diseases, with annual variability in medical costs. Pediatric MS and NMO are extremely rare in South Korea, with significant regional disparity in healthcare utilization. The findings emphasize the need for targeted healthcare policies to improve access and reduce disparities, particularly for chronic and rare diseases requiring specialized care.

## 1. Introduction

Multiple sclerosis (MS) and neuromyelitis optica (NMO) are chronic acquired demyelinating syndromes (ADS) of the central nervous system. These ADS can present with various symptoms, such as visual loss, sensory disturbances, weakness, difficulties in coordination, or bladder dysfunction. Over 50% of children with an incident demyelinating attack have a monophasic illness [1]. It is important to evaluate the pattern of disease activities with a radiographic feature for the differential diagnosis.

The prevalence of MS and NMO vary according to age, sex, ethnicity, and study period. The incidence and prevalence of MS in Western countries is much higher than that in Asian countries [2]. In South Korea during 2010–2017, the incidence and prevalence of MS is reported as 0.5 and 3.23 per 100,000 persons, retrospectively [2,3,4]. A recent systematic review reported the annual incidence and prevalence of NMO in South Korea were found to as high as those in Western countries [4,5,6]. The incidence and prevalence of NMO is reported as 0.41 and 3.36 per 100,000 population. MS and NMO usually affects young adults aged between 20 and 40 years and the proportion of pediatric cases initiating before 18 years of age is relatively small [4,7]. Pediatric-onset accounted for 1.6–9.5% of all MS cases and 3–5% of all NMO cases [6,8]. Epidemiological data on pediatric MS and NMO remains largely unknown worldwide. It is estimated that the epidemiology in children will demonstrate similar geographic patterns to that observed in adults.

Like adults, children with ADS face continuous disease status monitoring and need to periodic and long-term medical visits with pediatric neurology specialists. However, their healthcare utilization patterns and economic burden related to chronic diseases are not currently well understood. Therefore, it is essential to deeply investigate the healthcare-seeking behaviors of children with ADS. By closely examining their patterns of healthcare utilization, we can gain valuable insights into how to tailor medical strategies and policies to meet the needs of children with rare diseases and chronic conditions. Such insights are crucial for ensuring high-quality care access and optimizing outcomes for these vulnerable pediatric populations.

In this study, we used a nationwide population-based database of Health Insurance Review and Assessment Service (HIRA) to identify incident and prevalent pediatric MS and NMO cases. The primary outcome was to demonstrate the incidence and prevalence of pediatric MS and NMO in South Korea. The second objective was to demonstrate the pattern of healthcare utilization by geographic region in patients with MS and NMO.

## 2. Materials and Methods

### 2.1. Data Source

This retrospective study used data retrieved from the Korean HIRA database (Dataset No. M20210210108). The HIRA database covers 98% of the Korean population and provides healthcare claims data of outpatients and inpatients, such as demographics, diagnostic codes, dates and types of hospital visits, procedures, and prescription records [9]. The diagnostic codes used in the HIRA database are based on the International Classification of Disease, 10th revision (ICD-10). In addition, the HIRA database provides information about rare intractable disease (RID) registration. This RID system is set by the Rare Disease Management Act, in which the government subsidizes medical expenses for patients with rare diseases through a co-payment assistance policy. Registration of RIDs requires a physician-issued certificate that the patient meets the RID diagnostic criteria for a rare disease.

### 2.2. Study Population

We identified pediatric MS and NMO cases (age < 20 years) from 1 January 2016, to 31 December 2020. MS and NMO are rare ADS registered in the RID system. For incidence, the date of the first claim with registration to the RIDs was defined as the index date. Incidence cases were defined when patients were registered with V022 for MS or V276 for NMO in the RID at the index date. Prevalent cases were defined when those assigned the RID code had claim data with G35 for MS or G36 for NMO in ICD-10 as a primary and secondary diagnosis.

### 2.3. Statistical Analyses

Demographics and characteristics are presented as means with standard deviations or frequencies. The age-standardized incidence was calculated based on the mid-year population of those under the age of 20 from the Korean Statistical Information Service. We compared the annual standardized incidence and prevalence using the corresponding 95% confidence intervals (CIs). Standardized incidence rate ratios (IRRs) were applied, and IRRs were comparatively analyzed based on 2020. We estimated the annual direct medical costs of both inpatient and outpatient services. We counted the frequency of healthcare utilization, including outpatient clinics, emergency room visits, and hospitalizations. We calculated the percentage of healthcare utilization according to the regional districts and visualized the patterns of healthcare utilization on a geographic map of South Korea with color gradients. The economic burden after the index date was estimated as insurance-covered medical costs by summing hospitalizations, outpatient clinics, and pharmacies. Insurance-covered medical costs were divided by the number of prevalent cases and presented as annual medical costs per patient. The medical costs were converted into yearly currencies. Statistical significance was set at *p* < 0.05. Statistical analyses were performed using SAS (version 9.4; SAS Institute, Cary, NC, USA) and R version 4.0.5 (R Foundation for Statistical Computing, Vienna, Austria).

## 3. Results

### 3.1. Epidemiology of Pediatric MS and NMO

1. We identified 56 incident and 277 prevalent MS cases (Table 1). These prevalent cases had a female-to-male ratio of 1.94:1. The median age of onset of pediatric MS was 16.5 years (interquartile range [IQR] 10.5–18). The age-standardized incidence of pediatric MS was 0.21 (95% CI, 0.13–0.33) in 2016 and dropped to 0.06 (95% CI, 0.02–0.13) in 2020. Notably, the age-standardized incidence in 2016 and 2017 was significantly higher, with IRRs of 2.77 (95% CI, 0.99–7.69) and 3.61 (95% CI, 1.34–9.73), respectively, compared to that of 2020. The prevalence of pediatric MS in 2020 was 0.55 (95% CI, 0.41–0.73) per 100,000 persons, and this prevalence rates remained stable throughout the study period.
2. There were 34 incident and 140 prevalent NMO cases from 2016 to 2020 (Table 2). Similarly to pediatric MS, there was a female predominance in prevalence (a female-to-male ratio of 2.26). The median age was 15.5 years (IQR 11–18). The incidence and prevalence of pediatric NMO were much lower than those of MS that standardized IR of NMO was 0.07 (95% CI, 0.03–0.15) in 2016 and dropped to 0.03 (95% CI, 0.01–0.1) in 2020.

### 3.2. Pattern of Healthcare Utilization and Medical Costs

**Figure 1:**
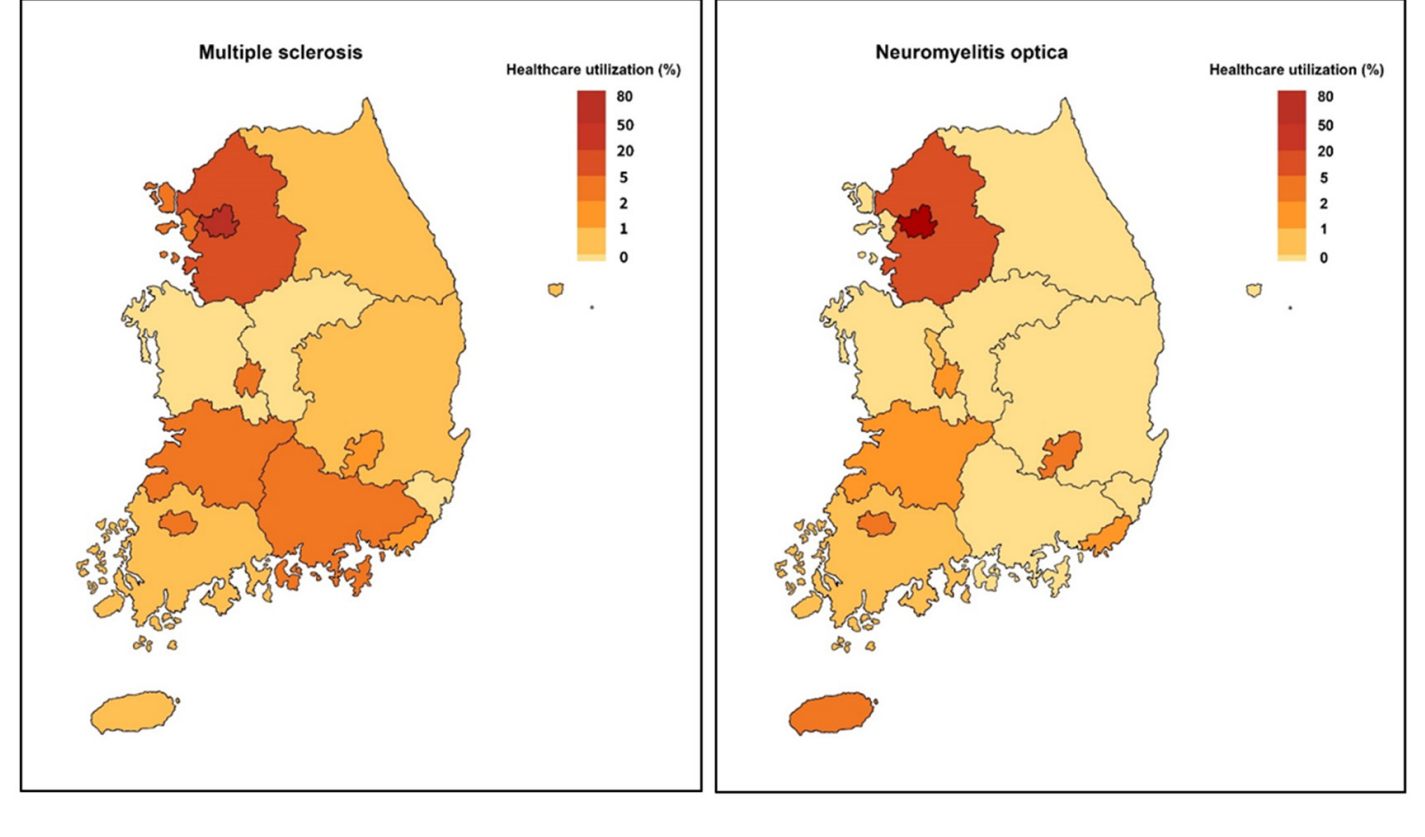
Gradient map displaying healthcare utilization pattern in patients with multiple sclerosis and neuromyelitis optica (2016–2020). Patients with multiple sclerosis and neuromyelitis optica mainly utilized healthcare located in Seoul and metropolitan area.

In prevalent cases of MS and NMO, about 70% of them visited tertiary hospitals. Most healthcare service was provided though outpatient visits. They rarely visited the emergency room, and the annual number of admissions per person were 0.5–0.9 cases per year in MS and 1.0–2.3 cases per year in NMO (Table 2). The total direct medical cost per person per year was USD 4631 for MS and USD 2961 for NMO in 2020. There is a distinctive geographic disparity in healthcare utilization. About 79.5% of MS cases and 86.5% of NMO cases utilized healthcare located in Seoul and metropolitan area of Gyeonggi-do, retrospectively (Figure 1). Healthcare utilization of other districts in patients with MS and NMO was less than 5.0%.

## 4. Discussion

This study effectively utilized a nationwide population-based database to explore the epidemiology and healthcare utilization associated with pediatric MS and NMO in South Korea. Both conditions were rare in the pediatric population, with incidence and prevalence rates significantly lower than those reported in Western countries. Furthermore, there is a distinct imbalance in healthcare utilization, heavily skewed towards urban areas, particularly Seoul and metropolitan regions. This geographic disparity highlights the need for more accessible healthcare services across all regions, especially in rural areas, to ensure equitable healthcare access for all patients regardless of location. Moreover, the economic analysis suggests that the direct medical costs associated with MS and NMO are subject to significant annual fluctuations, underscoring the financial volatility faced by families of patients with these rare diseases.

### 4.1. Rarity of Pediatric ADS

Similar to global epidemiology, the proportion of pediatric ADS, MS and NMO, in Korea is also much smaller than that of adults [4,6,7]. The prevalence of pediatric MS in Europe and the United States was much higher than that in Asia, which is concordant with the whole MS prevalence [3,8]. In Asia Pacific region, it was found to be variable, ranged from 0.69 to 26.2 per 100,000 persons [10,11]. Our prevalence of pediatric MS (0.55–0.63/100,000 in age < 20 years, 2016–2020) was comparable to that in Japan (0.69/100,000 in age ≤ 15 years, 2005–2007) and Taiwan (0.91–1.35/100,000 in age < 20 years, 2001–2015) [10,12]. Comparatively, based on past data, it has been observed that the prevalence of MS was higher in Western Asian regions such as Kuwait (1.3–6.3/100,000 in age < 18, 1994–2013), unlike in the Southeast Asia region [13]. The incidence of pediatric MS in our study (0.06–0.21/100,000 in those age < 20 years, 2016–2020) was lower than that reported in previous epidemiologic studies in Korea (0.23/100,000 in those age ≤ 17 years, 2010–2017) [14]. While the incidence of pediatric MS in Korea is decreasing, past incidences in other countries in Asia showed a somewhat higher trend, as follows: Taiwan (0.39–0.71 in age < 20 years, 2003–2015), and Kuwait (0.3–2.1 in age < 18 years, 1994–2013) [10,13]. Therefore, follow-up studies are needed in each country to determine if the decreasing trend in pediatric MS incidence is also evident.

Pediatric NMO exhibits much lower incidence and prevalence rates compared to MS [15]. Our annual incidence rate of pediatric NMO was 0.09/100,000 from 2016 to 2020, consistent with findings from a previous Korean epidemiological study (0.09/100,000 from 2010 to 2017) and a study from Taiwan (0.11/100,000 from 2001 to 2015) [10,14]. The incidence of pediatric NMO itself is rare, and its prevalence has remained stable over time. The female-to-male ratio in MS and NMO is well-documented as showing female predominence [7,15]. Our data align with these findings, indicating a higher prevalence among female in cases of MS (1.94:1) and NMO (2.26:1). Additionally, reports suggest that female predominance becomes more evident post-puberty in pediatric cases by puberty hormonal changes [7].

### 4.2. Diagnosis of ADS

MS diagnosis follows the 2010 revised McDonald criteria, which require clinical attacks and clinical evidence based on magnetic resonance imaging (MRI) lesions to determine lesion dissemination in space and time [16]. The 2017 revised McDonald criteria emphasized the role of cerebrospinal fluid (CSF) analysis and also considered asymptomatic MRI lesions in MS diagnosis [17].

Unlike MS, NMO diagnosis is facilitated by the presence of anti-aquaporin-4-IgG (AQP4-IgG). A diagnosis of NMO is made with a positive test for AQP4-IgG and at least one core clinical characteristic [18]. In the absence of AQP4-IgG, two or more core clinical characteristics and additional MRI criteria are required. When differentiating from MS, we should consider the location (such as the dorsal medulla/area postrema, periependymal regions in the brainstem, diencephalic structures, or cerebral hemisphere) and the shape (large, confluent, or tumefactive cerebral lesions) of MRI lesions. Nonetheless, due to inherent limitations in these distinguishing characteristics, it is important to consider clinical features and clinical course.

Recent data indicate a decline in the annual age-standardized incidence rate of pediatric MS in Korea, from 2010 to 2020, while the incidence among adults has remained relatively stable (0.53 in 2010 and 0.51 in 2016) [4]. This decrease in pediatric cases may be attributed, in part, to increased recognition of myelin oligodendrocyte glycoprotein-associated disorder (MOGAD) among pediatric neurologists, spurred by pivotal studies published in 2014 [19]. The growing awareness of MOGAD in recent years has elevated MOG antibody testing to a critical diagnostic tool, essential for differentiating between MS, NMO, and MOGAD, particularly in patients presenting with abnormal neurology and radiology including optic neuritis and myelitis. The widespread implementation of MOG antibody testing has significantly enhanced the accuracy of differential diagnoses across these conditions. However, MOGAD is not currently classified within the ICD-10 diagnostic codes, therefore, it is uncertain how the emergence of MOGAD impacts the incidence of MS. Future research on this issue is necessary.

### 4.3. Healthcare Utilization and Economic Burden of Pediatric ADS

Our study reveals the typical imbalance in healthcare utilization that are observed in ADS patients in Korea. According to the data of healthcare utilization, about 80% of patients received medical treatment in capital city, Seoul, and the medical utilization rate for this disease in areas outside of Seoul and the capital regions is less than 5%. Considering that Seoul accounts for only 0.6% of the entire territory of South Korea, it is evident that there is a concentration of medical service utilization in capital city, Seoul. Similarly, Yoon et al. also mentioned the notable geographic concentration of demyelinating diseases in medical centers within Seoul and its metropolitan areas [14].

Rare disease epidemiology from population-based study is crucial for public health planning [20]. In Korea, the imbalance tended to be concentrated within tertiary hospitals that possess expertise in pediatric neurology. Our data could provide evidence for establishing healthcare policies aimed at reducing disparities in medical service utilization across different regions. Healthcare policies to ensure balanced nationwide access to medical services for children with rare diseases are needed. With such national supports, local healthcare providers could more effectively diagnose pediatric rare diseases, ensuring timely medical treatment for affected children.

Medical costs of rare diseases are influenced by several factors, including diagnostic delay, the severity, the accessibility of healthcare resources, and practice patterns in each country [21]. It is difficult to directly compare economic burden of pediatric ADS across different countries and within different time frames. South Korea has the mandatory National Health Insurance system which is a public and single payer system [22]. This compulsory insurance is operated by the Korean government and provides insurance benefits regarding medical services. Most of the medical services which healthcare provides are reimbursed by the Korean government that Korean government implemented several policies to control medical expenditures. That’s why the medical costs from the Korean patients are lesser than those in US and Canada. Minegishi et al. demonstrated the increasing trend of hospital utilization and costs by pediatric ADS in the US from 2003 to 2012 [23]. It emphasized the growing economic and medical burden in ADS in pediatric healthcare, necessitating enhanced resources. However, our data, which calculated the direct medical costs per person from 2016 to 2020, showed that the medical expenses have not increased. The five-year study period is relatively short to determine trends of increase or decrease, and there was also annual variability in the length of stay per admission, which can affect medical costs.

### 4.4. Limitations

This study had several limitations. First, this study adopted working definitions used in previous nationwide database studies. We identified ADS patients registered for co-payment assistance for RID. This could be both an advantage and a disadvantage of a papulation-based study. An example of potential inaccuracy in the data is that the incidence estimates might be very conservative because only patients who received certificates based on the comprehensive medical evaluation according to the diagnostic criteria were included. Conversely, we could not validate or verify cases based on clinical data (MRI, CSF profile), so we cannot rule out the possibility that some MOGAD patients were included. Second, the HIRA database does not provide the permanent address of each patient, so we could not determine regional prevalence rates in Korea. We distinguished the locations where the medical practices were established, deriving a regional imbalance where medical utilization is concentrated more in urban than rural area.

## 5. Conclusions

In conclusion, our study underscores the necessity for targeted healthcare policies that address both the regional disparities in service availability and the economic burden associated with rare pediatric diseases. By improving healthcare access and affordability, particularly through the development of specialized services in underrepresented regions, we can better support the health and well-being of this vulnerable population. Continued research and policy efforts are crucial to ensure that all children with rare diseases receive timely and effective treatment, ultimately contributing to a more balanced healthcare system.

## Figures and Tables

**Table 1:** Annual incidence and prevalence rate of pediatric multiple sclerosis in South Korea (2016–2020).

Multiple Sclerosis				
Year	Standardized IR (95% CI)	Standardized IRR (95% CI)	IRR, *p*–Value	Standardized PR (95% CI)
2016	0.21 (0.13–0.33)	3.61 (1.34–9.73)	0.007	0.61 (0.46–0.80)
2017	0.16 (0.09–0.27)	2.77 (0.99–7.69)	0.041	0.63 (0.48–0.82)
2018	0.11(0.06–0.2)	1.85 (0.62–5.5)	0.259	0.63 (0.48–0.83)
2019	0.07 (0.03–0.15)	1.16 (0.35–3.85)	0.803	0.55 (0.41–0.74)
2020	0.06 (0.02–0.13)	Reference	–	0.55 (0.41–0.73)
**Neuromyelitis optica**				
**Year**	**Standardized IR (95% CI)**	**Standardized IRR (95% CI)**	**IRR, *p*–value**	**Standardized PR (95% CI)**
2016	0.07 (0.03–0.15)	2.07 (0.52–8.22)	0.289	0.15 (0.09–0.27)
2017	0.09 (0.05–0.18)	2.7 (0.72–10.15)	0.126	0.24 (0.15–0.37)
2018	0.07 (0.04–0.16)	2.18 (0.56–8.55)	0.252	0.31 (0.21–0.46)
2019	0.09 (0.04–0.18)	2.59 (0.68–9.82)	0.146	0.38 (0.26–0.53)
2020	0.03 (0.01–0.1)	Reference	–	0.41 (0.29–0.58)

Abbreviations: IR, incidence rate; IRR, incidence rate ratio; PR, prevalence rate; CI, confidence interval.

**Table 2:** Annual healthcare utilization in patients with multiple sclerosis and neuromyelitis optica (2016–2020).

Multiple Sclerosis					
	2016	2017	2018	2019	2020
Prevalent case, n	60	60	59	50	48
Type of healthcare, n (%)					
Tertiary hospital	41 (68.3)	43 (71.7)	41 (69.5)	36 (72.0)	37 (77.1)
General hospital	17 (28.3)	16 (26.7)	16 (27.1)	13 (26.0)	11 (22.9)
Others	2 (3.3)	1 (1.7)	1 (3.4)	1 (2.0)	0 (0)
Outpatient visits, n (SD)	7.1 (8.5)	7.3 (10.2)	7.00 (5.6)	6.4 (6.2)	6.7 (6.2)
ER visits, n (SD)	0.1 (0.4)	0.0 (0.2)	0.0 (0.2)	-	0.1 (0.5)
Admissions, n (SD)	0.9 (1.1)	0.7 (1.1)	0.6 (1.0)	0.5 (0.7)	0.6 (0.7)
Length of stay per admission, days (SD)	5.3 (15.3)	4.6 (13.4)	4.9 (12.9)	2.3 (4.3)	2.4 (3.8)
Direct medical costs, USD ^#^, mean	5778	5688	7291	5338	4631
**Neuromyelitis optica**					
Prevalent case, n	15	26	29	34	36
Type of healthcare, n (%)					
Tertiary hospital	11 (73.3)	20 (76.9)	22 (75.9)	30 (88.2)	33 (91.7)
General hospital	3 (20.0)	6 (23.1)	6 (20.7)	4 (11.8)	3 (8.3)
Others	1 (6.7)	0 (0)	1 (3.4)	0 (0)	0 (0)
Outpatient visits, n (SD)	3.2 (3.0)	7.6 (5.9)	7.5 (8.4)	7.8 (8.5)	8.2 (7.3)
ER visits, n (SD)	0.1 (0.4)	0.1 (0.3)	0.3 (1.2)	0.1 (0.4)	0.0 (0.2)
Admissions, n (SD)	1.3 (1.8)	2.3 (2.7)	1.2 (2.0)	1.0 (1.5)	1.5 (2.3)
Length of stay per admission, days (SD)	12.3 (15.4)	15.6 (25.8)	10.7 (25.4)	6.4 (10.7)	5.4 (8.7)
Direct medical costs per person, USD ^#^, mean	3881	5524	5673	3133	2961

Abbreviation: SD, standard deviation; ER, emergency room; KRW, South Korean won; USD, United States dollar. Data shown based on the person per year. ^#^ Currency expressed by the Korean economic statistics system: 2016 USD (1 USD = 1161 KRW), 2017 USD (1 USD =1131 KRW), 2018 USD (1 USD = 1100 KRW), 2019 USD (1 USD = 1166 KRW), and 2020 USD (1 USD = 1180 KRW).

## Data Availability

Data from the Korean national HIRA database are not publicly available due to privacy concerns. Data are not disclosed to the public or allowed to be exported.

## References

[1] Wattjes M.P., Ciccarelli O., Reich D.S., Banwell B., de Stefano N., Enzinger C., Fazekas F., Filippi M., Frederiksen J., Gasperini C. (2021). 2021 MAGNIMS-CMSC-NAIMS consensus recommendations on the use of MRI in patients with multiple sclerosis. Lancet Neurol..

[2] Browne P., Chandraratna D., Angood C., Tremlett H., Baker C., Taylor B.V., Thompson A.J. (2014). Atlas of Multiple Sclerosis 2013: A growing global problem with widespread inequity. Neurology.

[3] Cheong W.L., Mohan D., Warren N., Reidpath D.D. (2018). Multiple Sclerosis in the Asia Pacific Region: A Systematic Review of a Neglected Neurological Disease. Front. Neurol..

[4] Kim J.E., Park S.H., Han K., Kim H.J., Shin D.W., Kim S.M. (2020). Prevalence and incidence of neuromyelitis optica spectrum disorder and multiple sclerosis in Korea. Mult. Scler..

[5] Bagherieh S., Afshari-Safavi A., Vaheb S., Kiani M., Ghaffary E.M., Barzegar M., Shaygannejad V., Zabeti A., Mirmosayyeb O. (2023). Worldwide prevalence of neuromyelitis optica spectrum disorder (NMOSD) and neuromyelitis optica (NMO): A systematic review and meta-analysis. Neurol. Sci..

[6] Lee H.L., Kim J.Y., Seok J.M., Hong Y.H., Lim N.G., Shin H.Y., Kim B.J., Hwang S.Y., Min J.H., Kim B.J. (2020). Prevalence and Incidence of Neuromyelitis Optica Spectrum Disorder in Korea: Population Based Study. J. Korean Med. Sci..

[7] Dell’Avvento S., Sotgiu M.A., Manca S., Sotgiu G., Sotgiu S. (2016). Epidemiology of multiple sclerosis in the pediatric population of Sardinia, Italy. Eur. J. Pediatr..

[8] Yan K., Balijepalli C., Desai K., Gullapalli L., Druyts E. (2020). Epidemiology of pediatric multiple sclerosis: A systematic literature review and meta-analysis. Mult. Scler. Relat. Disord..

[9] Kim J.A., Yoon S., Kim L.Y., Kim D.S. (2017). Towards Actualizing the Value Potential of Korea Health Insurance Review and Assessment (HIRA) Data as a Resource for Health Research: Strengths, Limitations, Applications, and Strategies for Optimal Use of HIRA Data. J. Korean Med. Sci..

[10] Lin W.S., Wang H.P., Chen H.M., Lin J.W., Lee W.T. (2020). Epidemiology of pediatric multiple sclerosis, neuromyelitis optica, and optic neuritis in Taiwan. J. Neurol..

[11] Eskandarieh S., Sahraiain M.A., Molazadeh N., Moghadasi A.N. (2019). Pediatric multiple sclerosis and its familial recurrence: A population based study (1999–2017). Mult. Scler. Relat. Disord..

[12] Yamaguchi Y., Torisu H., Kira R., Ishizaki Y., Sakai Y., Sanefuji M., Ichiyama T., Oka A., Kishi T., Kimura S. (2016). A nationwide survey of pediatric acquired demyelinating syndromes in Japan. Neurology.

[13] Alroughani R., Akhtar S., Ahmed S.F., Behbehani R., Al-Abkal J., Al-Hashel J. (2015). Incidence and prevalence of pediatric onset multiple sclerosis in Kuwait: 1994–2013. J. Neurol. Sci..

[14] Yoon H.H., Park J.Y., Kim S.Y., Lee N.M., Yi D.Y., Yun S.W., Lim I.S., Chae S.A. (2021). Epidemiology of Demyelinating Diseases in Korean Pediatric Patients. J. Child. Neurol..

[15] Hor J.Y., Asgari N., Nakashima I., Broadley S.A., Leite M.I., Kissani N., Jacob A., Marignier R., Weinshenker B.G., Paul F. (2020). Epidemiology of Neuromyelitis Optica Spectrum Disorder and Its Prevalence and Incidence Worldwide. Front. Neurol..

[16] Polman C.H., Reingold S.C., Banwell B., Clanet M., Cohen J.A., Filippi M., Fujihara K., Havrdova E., Hutchinson M., Kappos L. (2011). Diagnostic criteria for multiple sclerosis: 2010 revisions to the McDonald criteria. Ann. Neurol..

[17] Thompson A.J., Banwell B.L., Barkhof F., Carroll W.M., Coetzee T., Comi G., Correale J., Fazekas F., Filippi M., Freedman M.S. (2018). Diagnosis of multiple sclerosis: 2017 revisions of the McDonald criteria. Lancet Neurol..

[18] Wingerchuk D.M., Banwell B., Bennett J.L., Cabre P., Carroll W., Chitnis T., de Seze J., Fujihara K., Greenberg B., Jacob A. (2015). International consensus diagnostic criteria for neuromyelitis optica spectrum disorders. Neurology.

[19] Jarius S., Paul F., Aktas O., Asgari N., Dale R.C., de Seze J., Franciotta D., Fujihara K., Jacob A., Kim H.J. (2018). MOG encephalomyelitis: International recommendations on diagnosis and antibody testing. J. Neuroinflamm..

[20] Mazzucato M., Visona Dalla Pozza L., Manea S., Minichiello C., Facchin P. (2014). A population-based registry as a source of health indicators for rare diseases: The ten-year experience of the Veneto Region’s rare diseases registry. Orphanet J. Rare Dis..

[21] Gimenez-Lozano C., Paramo-Rodriguez L., Cavero-Carbonell C., Corpas-Burgos F., Lopez-Maside A., Guardiola-Vilarroig S., Zurriaga O. (2022). Rare Diseases: Needs and Impact for Patients and Families: A Cross-Sectional Study in the Valencian Region, Spain. Int. J. Environ. Res. Public Health.

[22] Bae S., Yi B.K. (2022). Development of eClaim system for private indemnity health insurance in South Korea: Compatibility and interoperability. Health Inform. J..

[23] Minegishi M., Takahashi T., Testa M. (2019). Pediatric acquired demyelinating syndrome (ADS) in inpatient hospital settings: The hospitalization rate, costs, and outcomes in the US. Mult. Scler. Relat. Disord..

